# The Radical Cure of Reducible Hernia, by Injection

**Published:** 1853-01

**Authors:** 


### *The Radical Cure of Reducible Hernia, by Injection.*
By John Watson, M. D.

I do not propose, just now, to go into the whole merits, or demerits, of this operation, a task which has been assigned to other hands, and which has already been in some degree achieved, by one of the committees of the American Medical Association (see their report in the Transactions for the session of May last.) My only object is to give the details of a single case treated in this way, in connection with such hearsay information as I have been able to collect concerning the operation in other quarters.

The procedure now under consideration, if I am not mistaken, was first brought into notice through an irregular channel, by a certain Industrialist, of New England. But the first notice I remember to have seen of it, was in Professor Pancoast's *Operative Surgery.* In July, 1848, my attention was for a second time called to this subject, by a gentleman under my care for the treatment of varicocele, who, a year or more previously, had undergone an operation for the cure of a reducible hernia, by what he described as a trifling process, and with complete success. He spoke of it as a simple puncture, which subjected him to very little uneasiness; and assured me that he was cognizant to the cure or relief of other individuals, who had been treated like himself, by a practitioner of Boston. I subsequently ascertained that the instrument with which this individual operates, had been prepared by a cutler of this city; but on inquiry I found that it had been patented by the operator, and was, consequently, to be used only by himself. As I had made inquiry for it, with a view of employing it on a case then in hand, I

could not but feel indignant that any practitioner claiming to belong to the regular profession, should have thus prostituted his noble calling to mercenary ends; but believing that no special form of patent instrument is essential for the making of a puncture, or for introducing an irritating fluid beneath the integuments through this, I attempted in my own way to get along without it, as in the following case:

Joseph A. Seavell, of Ohio, seaman, aged 31, was admitted into the New York Hospital, Nov. 24, 1851, with a large inguinal hernia, occupying the left side of the scrotum, which had been then protruding for several hours, and had resisted several well-directed efforts for reduction. The patient for the last four years had been occasionally troubled by the protrusion, but had never before been baffled in his efforts to reduce it; and by the use of a truss he had been able to follow his regular occupation. With some little trouble the tumor was reduced by taxis, soon after his admission; and on the 29th of November, having explained my object to the patient, and obtained his consent, I attempted to effect a radical cure of the hernia.

While the patient was lying on his back, with his scrotum and left spermatic cord drawn slightly toward the right side, and with the integuments over the left external abdominal ring slightly on the stretch, I introduced the point of a delicate bistoury through the integuments, directly down to the crest of the os pubis, the point of the instrument touching, without dividing, the lower termination of Poupart's ligament, and made to work freely in the loose tissue immediately in front of the ring, but without wounding the spermatic cord. Having made the puncture, and withdrawn the bistoury, the nozzle of a small syringe charged with tincture of cantharides, was introduced through the wound, and about a drachm of this fluid was injected into the bottom of the cut, the hand of an assistant, in the meanwhile, resting firmly over the inguinal canal to prevent any portion of the injected fluid from entering this, or passing through the sac into the abdomen.

The whole procedure was the work of a few seconds, and gave the patient little or no uneasiness. I next applied a compress and spica bandage, to keep the parietes of the inguinal canal in close apposition, and administered an anodyne, keeping the patient on his back, with directions to apply an evaporating lotion, should severe inflammatory symptoms supervene.

In a few minutes after the operation, he began to speak of pain from the injection. The sore became more troublesome, and extended for several inches in every direction, but was severest along the ascending track of the spermatic cord. He slept but little during the following night; but next morning the pain had subsided, a slight soreness, only, remaining in the part. The patient was at the same time suffering from chancres. I made the treatment of these the pretext for keeping him on his back with the compress and bandage applied as above, for several days. He spoke of no uneasiness from the operation after the second day. On the 12th of December he was walking about without his truss, and with no apparent tendency to a recurrence of the hernial protrusion. On the following day, being desirous to join his vessel, which was about to sail for South America, he requested his discharge, promising to write to me, and report the further progress of his case, should the swelling reappear—and, if possible, to report in person, at the close of his voyage. But, as yet, I have not heard of him.

The operation in this instance, had evidently a beneficial effect, and I am not certain that it may not have effected a permanent cure. I am not disposed to believe that any portion of the injected fluid entered the hernial sac; but by exciting inflammation around this within the column of the external ring, and the subsequent condensation of tissues, which is so apt to follow acute inflammation, we can readily imagine that this procedure may, now and then, effect an object we have hitherto sought in vain to effect by other and severer measures.—*N. Y. Medical Times.*

